# Resistance- and endurance-trained young men display comparable carotid artery strain parameters that are superior to untrained men

**DOI:** 10.1007/s00421-024-05598-w

**Published:** 2024-10-03

**Authors:** Ian Hornby-Foster, Cory T. Richards, Aimee L. Drane, Freya M. Lodge, Michael Stembridge, Rachel N. Lord, Hannah Davey, Zaheer Yousef, Christopher J. A. Pugh

**Affiliations:** 1 Cardiff School of Sport and Health Science, Cardiff Metropolitan University, Cyncoed Road, Cardiff, CF23 6XD UK; 2 Musgrove Park Hospital, Somerset NHS Foundation Trust, Taunton, UK; 3 Health and Wellbeing Academy, Faculty of Medicine, Health and Life Sciences, Swansea University, Swansea, UK; 4 Department of Cardiology, University Hospital of Wales, Cardiff, UK; 5 Centre for Cardiovascular Research, Innovation and Development, Cardiff Metropolitan University, Cardiff, UK; 6 Centre for Health, Activity and Wellbeing Research, Cardiff Metropolitan University, Cardiff, UK; 7 University Hospital Southampton NHS Foundation Trust, Southampton, UK

**Keywords:** Two-dimensional strain imaging, Carotid artery stiffness, Resistance-exercise, Endurance-exercise, Arterial health

## Abstract

**Purpose:**

Central arterial stiffness, a predictor of cardiovascular risk, attenuates with endurance-exercise in ageing populations. However, in young individuals, this effect is inconsistent and emerging evidence suggests resistance-exercise may increase arterial stiffness. Two-dimensional (2D)-Strain imaging of the common carotid artery (CCA) is more sensitive at detecting endurance-training induced alterations in CCA stiffness than conventional methods, but has not been used to examine CCA stiffness in young resistance-trained individuals. Therefore, we compared CCA 2D-Strain parameters at rest, during acute exercise and recovery between resistance-trained, endurance-trained, and untrained young men.

**Methods:**

Short-axis CCA ultrasound images were obtained from 12 endurance-trained [27yrs (95%CI; 24–29)], 14 resistance-trained [24yrs (23–26)] and 12 untrained [23yrs (22–24] men at rest, during isometric handgrip (IHG) exercise and recovery. 2D-Strain analysis quantified CCA peak circumferential strain (PCS) and systolic (S-SR) and diastolic (D-SR) strain rates. Conventional stiffness indices included aortic pulse-wave velocity, CCA *β*-stiffness (*β*_1_) and Petersons elastic modulus (*E*_p_).

**Results:**

Resting conventional stiffness indices were not different between groups (*P* > 0.05). Resting PCS and S-SR were comparable between resistance- [11.6% (10.6–12.5) and 1.46 s^−1^ (1.37–1.55), respectively] and endurance-trained [11.4% (10.7–12.2) and 1.5 s^−1^ (1.38–1.62)] men and superior to untrained men [9.5% (9.19–9.9); *P* < 0.004 and 1.24 s^−1^ (1.17 – 1.31); *P* < 0.018)]. Both trained groups displayed comparable reductions in PCS and S-SR during IHG, which returned to resting values during recovery (*P* < 0.001), whereas these parameters remained unchanged in untrained men. D-SR decreased during IHG in all groups (*P* < 0.001), but to a lesser extent in endurance-trained men (*P* < 0.023), whereas *β*_1_ and *E*_p_ increased to a similar magnitude in all groups and returned to resting values during recovery (*P* < 0.001).

**Conclusion:**

Resistance- and endurance-trained men display comparable CCA 2D-Strain parameters that are superior to untrained men, which contends previous reports that resistance-training increases CCA stiffness.

## Introduction

The common carotid artery (CCA) is a low-resistance central elastic conduit that plays an essential role in the regulation of cerebrovascular perfusion (Tarumi et al. 2013). The elastic composition of the extracellular matrix significantly influences the CCA’s ability to distend and recoil in response to pulsatile ejection, which is crucial for maintaining consistent cerebral blood flow throughout the cardiac cycle (Wagenseil and Mecham 2009). In the presence of ageing and/or arterial disease, alterations occur to the extracellular composition, such as degeneration of elastin fibres and accumulation of cross-linked collagen, connective tissue, and advanced glycation end-products, which promote arterial stiffness and impair the vessel’s ability to buffer pulsatile blood flow (Wagenseil and Mecham 2009). Consequently, increased central artery stiffness is associated with hypertension, end-organ damage and stroke (Vasan et al. 2019), and is considered an independent predictor of cardiovascular disease (CVD) and all-cause mortality (van Sloten et al 2014; Laurent et al. 2006).

It is widely accepted that habitual endurance-exercise elicits potent vascular benefits and reduces arterial stiffness in ageing populations (Tanaka et al. 2000; Seals et al. 2019). This has been evidenced in middle-aged and older endurance-trained individuals who possess reduced aortic pulse wave velocity (PWV), CCA *β*_1_-stiffness index (*β*_*1*_), and elevated central artery compliance, when compared to sedentary and even recreationally active age-matched individuals (Seals et al. 2019). Although this observation is less consistent in young individuals (Tanaka et al. 2000), there have been reports associating regular endurance-exercise with reduced central artery stiffness in younger adults (Pugh et al. 2018). The precise mechanisms through which endurance-training reduces central artery stiffness are not fully understood, but may, in part, relate to favourable alterations to the elastic properties of the extracellular matrix within the arterial wall (Seals 2014; Hanna et al. 2014). In contrast, irrespective of age, the influence of regular resistance-training on central artery stiffness appears to be far more varied (Tagawa et al. 2021). A meta-analysis by Miyachi et al. (2013) demonstrated that moderate and high-intensity resistance-training is associated with increased arterial stiffness in young individuals, with augmented PWV and CCA *β*_*1*_ reported. Indeed, whilst two of the analysed studies reported no effect of resistance-training, all other studies observed an increase in arterial stiffness following resistance-training, equating to a mean increase of 14.3% (Miyachi et al. 2013). Accordingly, this has led to a call for further research to examine the link between resistance-training and increased arterial stiffness and the potential impact this may have on future cardiovascular health (Miyachi et al. 2013).

Studies reporting increases in arterial stiffness with resistance-training have suggested maladaptations, such as reduced cardiovagal baroreflex sensitivity (Nakamura et al. 2021), increased sympathetic vasoconstrictor tone (Pratley et al. 1994) and wall hypertrophy (Miyachi et al. 2003), as potential causal mechanisms. However, an alternative explanation for this apparent increase in stiffness may relate to other entirely healthy adaptations that occur with resistance-training; including increased arterial lumen diameter, vascular smooth muscle content, and collagen volume (Black et al. 2016a, b; Souza et al. 2017; Miyachi et al. 2003), which can all evoke consequential increases in wave reflections (Nakamura et al. 2021; MacDougall et al. 1985). Indeed, these structural and extracellular adaptations have been previously observed in resistance-trained rats (Souza et al. 2017), and likely occur in order to transiently prioritise structural integrity over compliance during intense resistance exercise to prevent acute rupture (Wagenseil and Mecham 2009). Importantly, there are no reports of resistance-training eliciting any of the extracellular elastin maladaptations that are associated with unfavourable (age-related) increases in arterial stiffness (Joseph and Mitchell 2007). Whereas, there is some evidence to indicate that resistance-training can enhance overall wall elasticity by increasing elastin content alongside collagen volume (Souza et al. 2017). Accordingly, under resting conditions, when less than 10% of collagen fibres are recruited and elastin is primarily responsible for stress transfer across the arterial wall (Wagenseil and Mecham 2009), it is plausible that resistance- and endurance-trained individuals would display comparable augmented central artery compliance.

Indeed, it is likely that the structural remodelling and intricate shifts in elastin-collagen ratio that occur with resistance-training markedly differ from the unfavourable extracellular alterations seen with ageing. However, it appears that conventional measures of arterial stiffness, like PWV and *β*_*1,*_ may lack the sensitivity to differentiate between these discrete central artery adaptations. For instance, PWV relates to the rate a pressure wave propagates through the circulation, which can augment with increased wall thicknesses (Laurent et al. 2006); an adaptation that is inherent to both resistance-trained and ageing arteries via distinct extracellular alterations (Souza et al. 2017; Joseph and Mitchell 2007). Similarly, *β*_1_ is limited to 1-dimensional measurement of arterial distension and therefore does not account for regional heterogeneity in wall stress across the lumen circumference (Bjallmark et al. 2010), which may explain the varied *β*_1_ responses to both endurance- (Tanaka et al. 2000) and resistance-training (Kawano et al. 2006; Miyachi et al. 2004) in young individuals. Taken together, as *β*_1_ lacks the sensitivity to characterise circumferential wall stress and PWV cannot differentiate between age-related structural maladaptations and healthy arterial remodelling, it is plausible that reports of unfavourable elevated stiffness in resistance-trained individuals may be misleading. Accordingly, techniques with greater sensitivity and specificity are required to provide a more comprehensive profile of central artery stiffness in resistance-trained individuals.

Two-dimensional strain (2D-Strain) imaging has emerged as a valuable tool for detecting heterogeneous CCA wall motion, providing sensitive wall deformation characteristics and a comprehensive index of global circumferential wall stress (Bjallmark et al. 2010). Indeed, this technique quantifies the magnitude and rate of circumferential deformation (strain) of the CCA wall across the cardiac cycle, which offers a more sensitive characterisation of age- (Bjallmark et al. 2010) and disease-related (Park et al. 2012; Kawasaki et al. 2009) CCA stiffness than *β*_1_ and superior inter/intra-operator reliability (Bjallmark et al. 2010). Furthermore, we have previously shown that 2D-Strain can detect fitness-induced reductions in arterial stiffness in young men that *β*_1_ and PWV were unable to identify (Pugh et al. 2018). Therefore, 2D-Strain may be a valuable tool to provide new insight into the effects of resistance-training on CCA stiffness, and reveal distinctions between the intrinsic CCA mechanical properties of endurance- and resistance-trained groups. Moreover, incorporating a physiological stimulus that provokes surges in sympathetic nerve activity, blood pressure and CCA loading, may also help to unmask different training-specific CCA adaptations that may not be evident under resting conditions.

Isometric handgrip (IHG) exercise is an established challenge capable of evoking acute increases in blood pressure and arterial loading that has previously revealed group differences in CCA 2D-Strain parameters (Campbell et al. 2023). During acute exercise such as IHG, the extracellular control of the CCA transfers from elastin to collagen fibres and thereby transiently increase CCA stiffness in order to maintain vessel stability and distal perfusion. However upon cessation, when both CCA loading and pressure return to resting levels, extracellular control transfers back to elastin fibres, causing CCA compliance to be restored to resting levels (Wagenseil and Mecham 2009). Since both endurance- and resistance-training appear to offer favourable changes to the elastic composition of the extracellular matrix (Souza et al. 2017; Hanna et al. 2014), trained groups may display a more dynamic CCA response during IHG (i.e. initial transient surge in CCA stiffness) than untrained counterparts and, importantly, a greater efficiency in restoring *normal* resting CCA compliance immediately after IHG exercise.

The aims of this study were to assess CCA 2D-Strain parameters both at rest, in response to acute IHG exercise and recovery, in resistance-trained, endurance-trained, and untrained young men. Our hypotheses were as follows: (i) resting CCA 2D-Strain parameters would be elevated in both trained groups versus untrained groups, and trained groups would also elicit an augmented dynamic response during and immediately following IHG exercise; (ii) CCA 2D-Strain imaging would be able to detect training-induced differences in CCA stiffness that are otherwise undetected by conventional indices at rest and in response to IHG exercise.

## Methods

### Ethical statement

Ethical approval was provided by Cardiff Metropolitan University’s School of Sport and Health Sciences Research Ethics Committee (17/3/01S), and the study conformed to the Declaration of Helsinki (2008) except for registration in a database. All participants were informed of the methods and study design verbally and in writing before providing written informed consent.

### Study participants

Data collection for this study was conducted as part of a wider investigation examining cardiac-specific training adaptations in men (Dawkins et al. 2020). However, the present study addresses a distinct a-priori hypothesis that directly relates to central artery-specific training adaptations in young men. In order to control for the well-documented independent effects of age on central artery stiffness, and focus our analysis exclusively on *young* males, only participants aged ≤ 35 years were included in the analysis of the present study (*n* = 40) (Segers et al. 2007; Mattace-Raso et al. 2012; Scuteri et al. 2012). Two recordings displayed inadequate tracking of the wall-lumen interface (*n* = 1 endurance; *n* = 1 untrained), so were unsuitable for analysis. Accordingly, 38 young males were included in the study analysis, comprising of 12 endurance-trained (runners, cyclists, triathletes; [mean age, 95% confidence interval (CI); 27 years, 95% CI (24, 29)], 14 resistance-trained [24 years, 95% CI (23, 26)] and 12 untrained [23 years, 95% CI (22, 24)] men. Average weekly training distance for endurance-trained men was 51 km for runners, 101 km for cyclists and 202 km for triathletes. All resistance-trained men exclusively performed moderate-to-high intensity full-body resistance-training and did not engage in any structured aerobic exercise. Untrained men engaged in ≤ 150 min of structured physical activity per week (Bull et al. 2020). Exclusion criteria: the use of cardioactive drugs and prescribed medications; the use of performance-enhancing drugs; history of cardiovascular, musculoskeletal, metabolic, or renal disease; any contraindications to exercise, asthma and smoking.

### Study design

Participants visited the laboratory on two separate occasions. Participants were asked to abstain from alcohol, caffeine and vigorous physical activity for 24 h prior to each visit and were asked to fast for ≥ 6 h prior to testing for the second visit. The first visit involved the completion of health and training questionnaires, anthropometric measurements and acquisition of resting blood pressure. A strength assessment was performed with a one-repetition maximum (1RM) seated leg-press. After a minimum 30 min recovery period, an assessment of cardiorespiratory fitness was carried out via a peak oxygen consumption ($\dot{V}{\text{o}}_{{{\text{2peak}}}}$) incremental cycling test. For the second visit, participants rested supine for 10 min before PWV and central blood pressure were assessed. Subsequently, short-axis ultrasound images of the CCA were recorded at rest, during a three-minute IHG protocol at 40% of maximal voluntary contraction and after a one-minute recovery period.

### Exercise testing

The 1RM seated leg-press was performed on a commercially available leg-press machine (Linear Leg Press, Life Fitness, Ltd., Queen Adelaide, UK). The 1RM protocol for the 45°-inclined double leg press was determined according to the National Strength and Conditioning Association guidelines (Baechle 2008). Participants initially completed a 5 to 10 repetition warm-up against light resistance. After a 2 min rest period, the first attempt was performed using a load that was ~ 50% of the participants’ weight-predicted 1RM. Following a 3–5 min rest, participants repeated the exercise with an increased load. This process was repeated until participants could only perform a single repetition and required between three and five attempts to achieve the correct load. $\dot{V}{\text{o}}_{{{\text{2peak}}}}$ and peak power output (PPO) was determined using an incremental cycling test on an upright cycle ergometer (Lode Corival, Groningen, Netherlands). Exercise was started at 50W for both the resistance-trained individuals and the controls, at 120W for endurance-trained individuals, and was subsequently increased by 20W every minute until volitional exhaustion. Measurements of ventilatory gas exchange were obtained using a mask-based, breath-by-breath analyser (Jaeger, Oxycon Pro, Warwick, UK) and heart rate was measured using a Polar heart rate monitor (Polar Electro, RS400, Kemple, Finland). Peak oxygen uptake was defined as the highest $\dot{V}{\text{o}}_{\text{2}}$ achieved over a 30 s consecutive period.

### Aortic pulse wave velocity

In accordance with applanation tonometry guidelines, a high-fidelity micromanometer-tipped probe was used to obtain sequential ECG-gated pressure waveforms at the site of maximal arterial pulsation of the carotid and femoral arteries to calculate aortic PWV (Van Bortel et al. 2012). Central blood pressure was estimated by applying a validated transfer function (Van Bortel et al. 2012) to radial artery waveforms collected via the same probe (SphygmoCor; AtCor Medical, Sydney, NSW, Australia).

### Common carotid artery ultrasonography

Short-axis grey-scale cine loops of the right CCA were acquired on a commercially available high-resolution ultrasound system (Vivid q, GE Healthcare, Chalfont, UK), using a 12 MHz multi-frequency linear array probe. CCA images were recorded ~ 2 cm proximal to the carotid bifurcation over a minimum of five consecutive cardiac cycles. Cardiac cycles were measured continuously using a 3-lead ECG connected to the ultrasound machine. Frame rate (92.3 frames per second), imaging depth and probe orientation was standardised for all participants and all images were acquired by a single trained technician.

### Two-dimensional strain and conventional ultrasound image analysis

Speckle-tracking software quantifies vascular tissue motion throughout the cardiac cycle by automatically identifying speckles in the short-axis ultrasound image (Pugh et al. 2018; Bjallmark et al. 2010). To quantify arterial strain and strain-rate, a region of interest (ROI) was manually placed over the entire CCA wall circumference, ensuring accurate circumferential alignment with the lumen-wall interface. The ROI comprised six evenly distributed segments in which movement of speckles were tracked frame-by-frame throughout the cardiac cycle using a speckle-tracking algorithm inherent to the software (EchoPac Version 112, GE Vingmed Ultrasound, Horten Norway). The software (automatic verification) and operator (visual verification) both verified suitable positioning of the ROI for optimal wall tracking, whereby images included within the analysis demonstrated successful tracking across all six ROI segments. This generates strain and strain rate curves. Peak circumferential strain (PCS), systolic strain-rate (S-SR) and diastolic strain-rate (D-SR) were expressed as ‘global’ values reflecting the average values obtained between the six ROI segments of the CCA over three consecutive cardiac cycles; therefore representing circumferential motion of the entire CCA wall. PCS was identified as the greatest peak in the interpolated circumferential strain curve and represents the magnitude of arterial deformation. S-SR was identified as the largest positive peak in the strain-rate curve that occurred after the QRS-complex, and D-SR was defined as the largest negative peak on the strain-rate curve that occurred after the T-wave of the ECG.

CCA diameters were measured by obtaining an M-mode trace through the centre of the short-axis image. Systolic and diastolic diameters were defined as the maximal and minimal diameters during the cardiac cycle, respectively, and were measured from the leading edge of the intima–lumen interface of the anterior wall to the leading edge of the lumen–intima interface of the posterior wall.

To characterize local CCA stiffness, Peterson’s Elastic Modulus (*E*_p_; kilopascal [kPa]), *β*_1_ (arbitrary units [AU]), *β*_2_-stiffness-index (*β*_2_; AU) and distensibility (mmHg × 10^−3^) (the inverse of *E*_p_) were calculated. *β*_1_, *E*_p_ and distensibility are conventional measures of CCA stiffness and adjust changes in arterial diameter throughout the cardiac cycle for changes in distending pressure (Laurent et al. 2006). *β*_2_ relates PCS to distending pulse pressure (Oishi et al 2008). Increases in *β*_1_, *β*_2_ and *E*_p_ are associated with greater arterial stiffness (Laurent et al. 2006), conversely increases in distensibility indicate a greater magnitude of arterial distension per unit of pressure. Stiffness measures were calculated using the following formulae:

Distensibility = [(SD−DD)/(SBP−DBP)]/DD (mmHg × 10^−3^)

*β*_1_ = ln(SBP/DBP)/[(SD−DD)/DD] *(AU)*

*β*_*2*_ = ln(SBP/DBP/PCS) *(AU)*

*E*_p_ = (SBP−DBP)/[(SD−DD)/DD] *(kPa)*

where ln refers to the natural logarithm function, SBP and DBP relate to peripheral systolic and diastolic pressure respectively, and SD and DD relate to systolic and diastolic diameter respectively.

### Isometric hand-grip exercise

Participants performed three left-handed maximal voluntary contraction using a handgrip force transducer device (MLT004/D, ADInstruments, Oxford, UK), with the highest achieved value of the three maximal efforts used to calculate exercise intensity. After a 10 min period of supine rest, whilst still lying supine, participants performed IHG exercise at 40% of this maximum contraction for 3 min. The individualised target force zone and real-time contraction force was continuously displayed on a computer screen and visually monitored by both the participant and an assessor to ensure adherence to the prescribed intensity throughout the protocol. This IHG exercise protocol was used as it has been previously shown to elicit transient increases in systemic blood pressure and evoke arterial responses (Campbell et al. 2023). CCA ultrasound measurements were obtained at rest (resting), 1 min 30 s following the onset of contraction (mid), immediately prior to release of contraction (end), and 1 min after release (recovery). Beat-by-beat peripheral (brachial) blood pressure was recorded continuously throughout the protocol via finger plethysmography (FinometerPro, FMS, Groningen, Netherlands), and calibrated against manual brachial blood pressure measurements obtained at rest.

### Statistical analysis

In the absence of available data on the effects of resistance-training on 2D-Strain parameters, power analysis was conducted by sampling pooled data from our two previous studies examining the impact of regular endurance exercise on 2D-Strain parameters (Pugh et al. 2018; Talbot et al. 2020). A mean difference in PCS of 2% (Cohen’s *d* effect size = 0.76) was detected between endurance-trained and untrained young men. Accordingly, we estimated that a sample of 38 participants within a three-group cross-sectional study design would detect a 2% (*d* = 0.76) difference in PCS with 80% power at a two-sided 0.05 significance level. All data were checked and confirmed for normality of distribution using a Shapiro–Wilk test and visual inspection of histogram. A one-way analysis of variance (ANOVA) was performed to assess differences in resting parameters between the three groups. A two-factor, repeated-measures ANOVA was used to determine the main effects of training status, time and any interaction between these factors (training status x time) at baseline, during IHG exercise and upon recovery. Where a significant interaction was observed, *post-hoc* comparisons with Bonferroni corrections were conducted to identify significant differences among group mean values. Additionally, if group differences were observed at rest, analysis of covariance (ANCOVA) was conducted on the IHG data with resting values set as a covariate. All data were analysed using the statistical package SPSS (Ver.27 for Windows, Chicago, SPSS Inc) and presented as means and 95% confidence intervals (95% CI) unless otherwise stated. Statistical significance was set at *P* < 0.05.

## Results

### Participant characteristics

Participant characteristics are listed in Table 1. By design, the endurance-trained group had a greater *V̇*o_2peak_ and peak power output than resistance-trained and untrained groups (*P* < 0.001). Similarly, resistance-trained men had a greater 1RM (*P* < 0.001) compared to endurance-trained and untrained groups. There were no differences between groups for height, body fat percentage or free fat mass (*P* > 0.05). However, resistance-trained men had a significantly higher body mass and body mass index (*P* < 0.001) compared to the other groups; and untrained men had a significantly higher resting heart rate compared to resistance-trained men (*P* = 0.015).

**Table 1:** Resting characteristics of endurance-trained, resistance-trained and untrained men

Characteristics	Endurance-trained	Resistance-trained	Untrained	*P* value§
Age (years)	27 [24, 29]†	24 [23, 26]	23 [22, 24]	0.04
Height (cm)	180.1 [177.1, 183]	181 [177.9, 184.2]	180.2 [175.3, 185.1]	0.95
Body mass (kg)	75.5 [70.3, 79.7]	87.9 [83.8, 92]†*	75.2 [71, 79.3]	< 0.001
BMI (kg m^−2^)	23.2 [21.8, 24.2]	27.8 [26.9, 28.7]†*	23.3 [21.6, 24.9]	< 0.001
Body fat (%)	12.1 [9.1, 15]	12.6 [10.6, 14.6]	15 [11.55, 18.5]	0.43
FFM (kg)	65.6 [63.6, 68.4]	76.2 [65.7, 86.7]	63.2 [60, 66.3]	0.38
$\dot{V}{\text{o}}_{{{\text{2peak}}}}$ (ml.kg^−1^.min^−1^)	56.1 [50.1, 61.9]†	39.6 [37.3, 41.8]*	39.9 [37.3, 42.5]	< 0.001
PPO (W)	335 [313, 357]†	301 [281, 321]†	231 [215, 246]	< 0.001
1RM (kg)	263 [226, 300]	438 [417, 459]†*	222 [188, 256]	< 0.001
Training history (years)	4.6 [3.5, 5.6]	6.2 [4.8, 7.5]		0.08
Training frequency (session/wk)	7.2 [5.8, 8.5]†	5.1 [4.6, 5.5]†	0.7 [0.3, 1.0]	< 0.001
cSBP (mmHg)	101 [98, 103]	100 [97, 102]	99 [96, 101]	0.22
cDBP (mmHg)	72 [68, 75]	71 [67, 74]	69 [66, 71]	0.89
cPP (mmHg)	29 [27, 30]	28 [25, 30]	31 [26, 35]	0.34
cMAP (mmHg)	82 [78, 85]	81 [78, 83]	79 [75, 82]	0.41

Data are presented as means (95% confidence interval)

*BMI* body mass index, *FFM* free fat mass, $\dot{V}{\text{o}}_{{{\text{2peak}}}}$ peak oxygen consumption, *PPO* peak power output, *1RM* one repetition maximum, *cSBP* central systolic blood pressure, *cDBP* central diastolic blood pressure, *cPP* central pulse pressure, *cMAP* central mean arterial pressure

§Indicates one-way ANOVA

*Indicates *P* < 0.05 versus endurance group

†Indicates *P* < 0.05 versus untrained group

### Carotid artery parameters and peripheral and central haemodynamics at rest

**Fig. 1:**
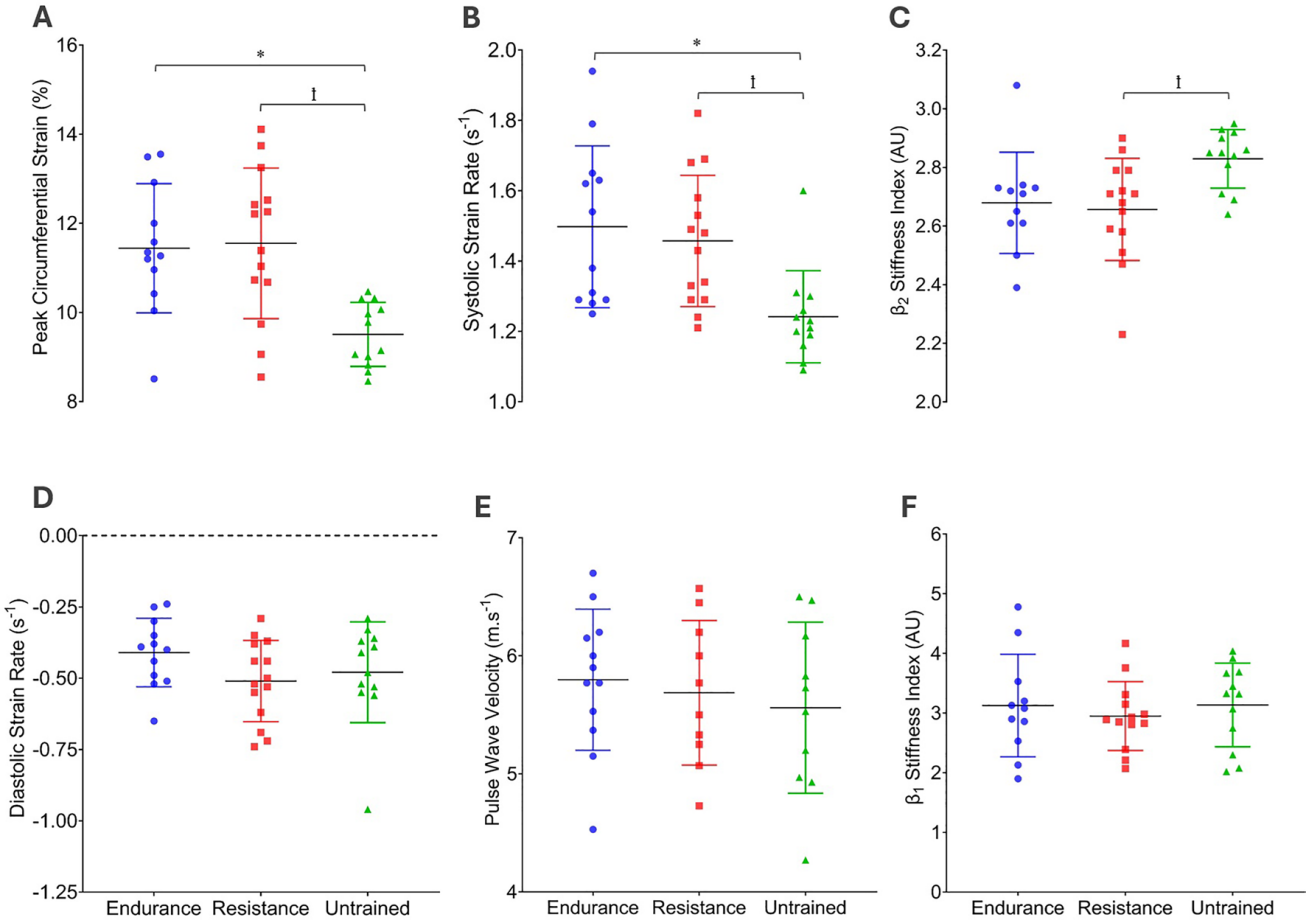
Resting peak circumferential strain (%); ANOVA *P* < 0.001 (**A**), systolic strain-rate (s^−1^); ANOVA *P* = 0.003 (**B**), *β*_2_ (AU); ANOVA *P* = 0.021 (**C**); Diastolic strain-rate (s^−1^); ANOVA *P* = 0.23 (**D**), aortic pulse wave velocity (m.s^−1^); ANOVA *P* = 0.67 (**E**), and *β*_1_ (AU); ANOVA *P* = 0.76 (**F**) in endurance-trained, resistance-trained and untrained men. Error bars represent SD. * Indicates *P* < 0.05 difference between endurance-trained vs. untrained groups. ꝉ Indicates *P* < 0.05 difference between resistance-trained vs. untrained groups

There were no differences in peripheral or central blood pressures between the three groups at rest, nor were there any group differences in PWV (*P* = 0.67; Fig. 1e) or *β*_1_ (*P* = 0.76; Fig. 1f). Similarly, there were no resting differences in CCA systolic (*P* = 0.89), diastolic (*P* = 0.76), or mean diameter (*P* = 0.76), distensibility (*P* = 0.83) or *E*_p_ (*P* = 0.41) between the three groups (Table 2). PCS and S-SR were elevated in resistance-trained (PCS: *P* = 0.002; S-SR: *P* = 0.018) and endurance-trained (PSC: *P* = 0.004; S-SR: *P* = 0.006) men compared to untrained men (Fig. 1a, b, respectively), whereas there were no differences in D-SR between the groups (Fig. 1d). *β*_2_ was elevated in untrained men compared to resistance-trained men, but not compared to endurance-trained men (*P* = 0.02; *P* = 0.15, respectively; Fig. 1c).

**Table 2:** Peripheral haemodynamics and common carotid artery parameters before, during and after a 3 min bout of isometric hand-grip exercise in endurance-trained, resistance-trained, and untrained men

Variable	Endurance-trained				Resistance-trained				Untrained			
	Rest	Midway	End	Recovery	Rest	Midway	End	Recovery	Rest	Midway	End	Recovery
Haemodynamics												
SBP (mmHg)	127 [120, 133]‡	136 [127, 144]‡	142 [131, 152]‡	127 [120, 133]‡	123 [118, 127]	138 [131, 144]‡	144 [138, 149]‡	130 [123, 136]‡	121 [117, 124]	130 [123, 136]‡	135 [125, 144]‡	122 [117, 126]
DBP (mmHg)	77 [71, 82]	83 [75, 90]‡	86 [77, 94]‡	75 [68, 81]	77 [72, 81]	90 [83, 96]‡	93 [87, 98]‡	80 [74, 85]	75 [71, 78]	83 [79, 86]‡	87 [79, 94]‡	76 [71, 80]
MAP (mmHg)	93 [87, 98]	105 [99, 110]‡	111 [102, 119]‡	92 [86, 97]	82 [77, 86]	104 [97, 110]‡	112 [105, 118]‡	91 [85, 96]	88 [84, 91]	102 [94, 109]‡	109 [99, 118]‡	91 [86, 95]
PP (mmHg)	50 [44, 55]	53 [47, 58]	56 [48, 62]	52 [46, 57]	47 [42, 51]	48 [43, 52]	51 [45, 56]	50 [44, 55]	46 [41, 50]	46 [41, 50]	48 [41, 54]	47 [41, 52]
HR (bpm)	57 [52, 61]	75 [70, 79]‡	81 [75, 86]‡	55 [50, 59]†	53 [45, 60]†	68 [58, 77]‡†	70 [57, 82]‡	52 [45, 58]†	67 [60, 73]	82 [72, 91]‡	81 [72, 89]‡	67 [60, 73]
CCA parameters												
Systolic diameter (mm)	6.28 [5.87, 6.68]	7.22 [6.84, 7.59]‡	7.34 [6.99, 7.68]‡	6.85 [6.49, 7.20]	6.35 [6.09, 6.60]	7.03 [6.67, 7.38]‡	7.17 [6.90, 7.43]‡	6.64 [6.40, 6.87]	6.43 [6.22, 6.63]	6.98 [6.81, 7.14]‡	6.95 [6.76, 7.13]‡	6.72 [6.60, 6.83]
Mean diameter (mm)	5.81 [5.44, 6.17]	6.72 [6.40, 7.03]‡	6.86 [6.54, 7.17]‡	6.39 [6.06, 6.71]	5.91[5.65, 6.16]	6.69 [6.39, 6.98]‡	6.86 [6.60, 7.11]‡	6.20 [5.98, 6.41]	6.0 [6.18, 6.39]	6.67 [6.51, 6.82]‡	6.62 [6.41, 6.82]‡	6.29 [6.18, 6.39]
Distensibility (mmHgx10-3)	3.46 [2.85, 4.06]	2.35 [1.64, 3.05]‡	1.69 [1.35, 2.02]‡	3.19 [2.45, 3.92]	3.61 [3.20, 4.01]	2.18 [1.58, 2.77]‡	1.94 [1.61, 2.26]‡	3.19 [2.47, 3.90]	3.50 [3.01, 3.98]	2.18 [1.71, 2.64]‡	2.42 [1.76, 3.07]‡	3.24 [2.74, 3.73]
Ep (kPa)	30 [26, 35]	47 [35, 59]‡	57 [59, 65]‡	34 [29, 39]	28 [25, 32]	46 [37, 55]‡	57 [47, 67]‡	36 [29, 43]	30 [26, 33]	53 [41, 65]‡	56 [36, 76]‡	33 [27, 39]

Data are presented as means (95% confidence interval)

*SBP* peripheral systolic blood pressure, *DBP* peripheral diastolic blood pressure, *MAP* peripheral mean arterial pressure, *PP* peripheral pulse pressure *HR* heart rate, *Ep* Peterson’s elastic modulus

‡Indicates P < 0.05 versus resting within the same group

†Indicates P < 0.05 versus untrained group at the same time-point

### Peripheral haemodynamics during handgrip exercise

Peripheral systolic, diastolic, pulse pressure and mean arterial blood pressures increased in response to the IHG protocol during the mid- and end-points and returned to resting values during recovery in all groups (Table 2; *P* < 0.001). However, no effects of training status (*P* > 0.24), or a time*training status interaction (*P* > 0.39), were observed on any of these variables. Heart rate significantly increased in response to the IHG protocol during the mid- and end-points and returned to resting values in all groups and a significant group (*P* = 0.03) and time*training status interaction effect (*P* = 0.04) was observed. Post-hoc pairwise comparisons revealed that heart rate was lower in resistance-trained compared to untrained men at the midpoint of the IHG exercise (*P* = 0.004). Whereas, both resistance-trained (*P* = 0.001) and endurance-trained (*P* = 0.007) men had a lower heart rate than untrained men during recovery.

### Carotid artery diameter and conventional stiffness indices during handgrip exercise

**Fig. 2:**
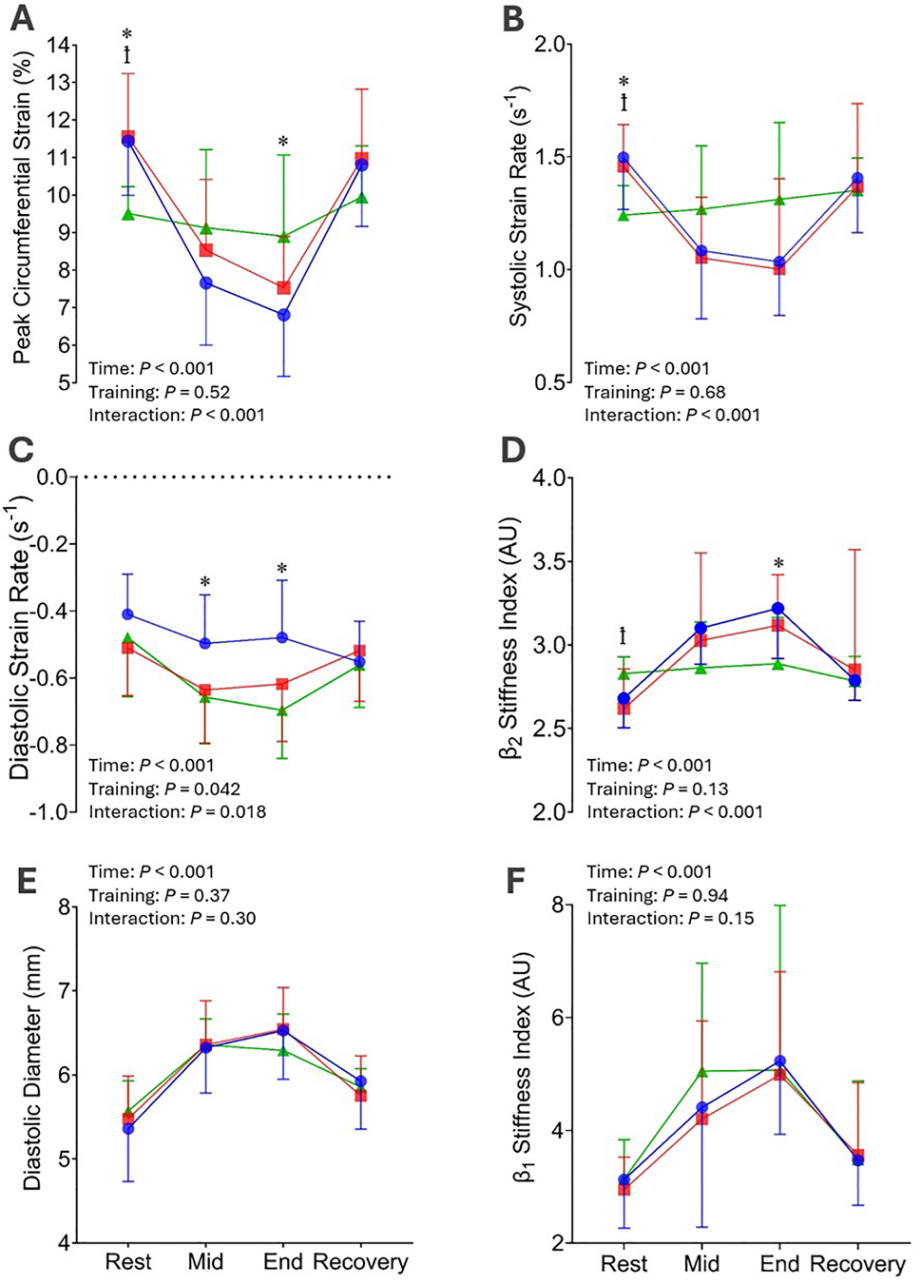
Peak circumferential strain (%) (**A**), systolic strain rate (s^−1^) (**B**), diastolic strain rate (s^−1^) (**C**), *β*_2_ (AU) (**D**), diastolic diameter (mm) (**E**) and *β*_1_ (AU) (**F**) before (“rest”), during (“mid” and “end”) and after (“rec”) a 3 min bout of isometric hand-grip exercise in resistance-trained, endurance-trained and untrained men. Error bars represent SD. *P* values for the two-way ANOVA data are presented (“time”, “training” and “interaction”). * Indicates *P* < 0.05 difference between endurance-trained vs. untrained groups at the same time-point. ꝉ Indicates P < 0.05 difference between resistance-trained vs. untrained groups at the same timepoint

A significant effect of time was observed in all CCA diameter and conventional stiffness variables (*P* < 0.001). In all groups, CCA diameter, *β*_1_, *E*_p_ increased, and distensibility decreased in response to the mid- and end-points of the IHG exercise and returned to resting values during recovery (Fig. 2 and Table 2; *P* < 0.001). No effects of training status (*P* > 0.37), or a time*training status interaction (*P* > 0.27), were observed on any CCA diameter or conventional stiffness variables (Fig. 2 and Table 2).

### Carotid artery 2D-strain parameters during handgrip exercise

A significant effect of time and time*training status interaction was observed for PCS and S-SR (Fig. 2a, b; *P* < 0.001). PCS and S-SR both decreased during the mid- and end-points of the IHG exercise in resistance-trained and endurance-trained men and returned to resting values during recovery. In contrast, no changes to PCS (*P* > 0.57), or S-SR (*P* > 0.65) were observed in the untrained group at any stage of the IHG protocol. During the IHG protocol, the reduction in PCS was significantly greater in the endurance-trained group than in the untrained group at the end stage of the protocol (*P* = 0.017; Fig. 2a), whereas no group differences were observed in S-SR at any stage during IHG exercise (*P* > 0.68; Fig. 2b). Significant main effects of time, training status and time*training status interaction were observed for D-SR. D-SR decreased during the IHG protocol in all groups (*P* < 0.001), with the magnitude of reduction being significantly less in endurance-trained men compared to resistance-trained and untrained men at both the mid (*P* = 0.023 and *P* = 0.012 respectively) and end (*P* = 0.038 and *P* = 0.003 respectively; Fig. 2c) stages of the protocol. Finally, significant effects of time and time*training status interaction were observed for *β*_2_ (*P* < 0.001). During the mid- and end-points of the IHG protocol, *β*_2_ increased in both trained groups and returned to resting values during recovery, but remained unchanged in the untrained group at each stage of the IHG protocol (*P* > 0.71). At the endpoint of the protocol, *β*_2_ was significantly higher in endurance-trained men compared to untrained men (*P* = 0.011; Fig. 2d), but this group difference disappeared by the recovery stage.

### Analysis of covariance

All significant time*training status interactions for PCS, S-SR and *β*_2_ throughout the IHG protocol remained after covariate adjustment for resting data (*P* = 0.005, *P* = 0.012 and *P* < 0.001, respectively), as did all significant *post-hoc* group differences.

## Discussion

The aim of this study was to assess CCA 2D-Strain parameters at rest and in response to IHG exercise in resistance-trained, endurance-trained, and untrained men. In line with our primary hypotheses, our main findings were as follows: (i) both trained groups demonstrated significantly elevated PCS and S-SR at rest compared to untrained controls; (ii) PCS and S-SR reduced during the IHG exercise to a similar magnitude in both trained groups, before quickly returning to resting levels during recovery; whereas untrained men demonstrated no changes in either parameter throughout the protocol; (iii) 2D-Strain parameters were able to detect differences in global CCA stiffness both at rest and during IHG exercise that were not identified with conventional stiffness indices. To our knowledge, this is the first study to utilise 2D-Strain imaging to demonstrate that resistance- and endurance-trained men display comparable global CCA compliance that is superior to untrained men. These findings indicate that both endurance and resistance-training offer potent arterial benefit and contends previous reports that resistance-training increases central artery stiffness.

### The influence of training status on carotid artery 2D-strain parameters at rest

Arterial stiffness is attenuated with regular endurance-training in middle-aged and older individuals (Tanaka et al. 2000; Seals et al. 2019), however this is not consistently replicated in young endurance- (Tanaka et al. 2000) or resistance-trained individuals (Rakobowchuk et al. 2005). Furthermore, resistance-training has been associated with increased central arterial stiffness in young and middle-aged individuals (Miyachi et al. 2013). Together, this has led to the proposition of a possible ceiling effect in young endurance-trained individuals (Montero et al 2017), and rising ambiguity over the relevance of increased arterial stiffness to the cardiovascular health of resistance-trained individuals (Miyachi et al. 2013). However, the present study demonstrates comparable PCS and S-SR values in young endurance- and resistance-trained men that are superior to age-matched untrained counterparts. Augmented resting PCS and S-SR likely reflect training-induced improvements in CCA wall elasticity and an enhanced capacity to buffer pulsatile flow, thereby ensuring smooth, consistent blood flow to the cerebral circulation (Tomoto et al 2015). This finding supports our previous observation of elevated resting PCS and S-SR in highly-fit young men compared to both moderately-fit (Pugh et al. 2018) and inactive counterparts (Talbot et al. 2020), countering suggestions of a ceiling effect with endurance-training in young men. Furthermore, superior PCS and S-SR values in both trained groups also challenge previous suggestions that resistance-training increases arterial stiffness (Miyachi et al. 2013). In fact, when PCS is presented relative to distending pulse pressure via *β*_2_ (Oishi et al. 2008), CCA stiffness is significantly lower in resistance-, but not endurance-trained, men compared to untrained men. Given that both PCS and S-SR are independent predictors of atherosclerotic development and CVD risk (Kawasaki et al. 2009), the findings of the present study suggest that sustained endurance and resistance-training offer comparable cardio-protection in young men. Accordingly, contrary to some previous reports (Miyachi et al. 2003; Miyachi et al. 2013; Okamoto et al. 2009), these data demonstrate that resistance-trained men possess healthy and compliant CCAs that are comparable to endurance-trained men and superior to untrained men.

Unlike 2D-Strain parameters, conventional indices of arterial stiffness, including PWV, *β*_1_ and *E*_p_, were unable to distinguish between the discrete groups, which brings into question the sensitivity of conventional measures to detect training-induced adaptations to central elastic arteries. Indeed, one-dimensional *β*_1_ and *E*_p_ indices do not consider global variations in arterial compliance, which limits their sensitivity to detect intricate alterations in CCA stiffness (Campbell et al. 2023; Pugh et al. 2018; Bjallmark et al. 2010). In contrast, 2D-Strain parameters reflect heterogenous arterial motion patterns across the entire wall circumference, thereby providing a more sensitive and detailed profile of global arterial wall stress and compliance variation (Kawasaki et al. 2009; Bjallmark et al. 2010). It is likely that this relative lack of sensitivity accounts for the disagreement between *β*_1_ and *E*_p_ with PCS in the present study, and may also explain previous reports of varied *β*_1_ responses to both endurance- (Tanaka et al. 2000) and resistance-training (Miyachi et al. 2013) in young individuals. It is also possible that previous reports of increased *β*_1_ and PWV in resistance-trained men are a consequence of increases in arterial diameter, wall thickness and collagen volume across the arterial tree (MacDougall et al. 1985). This may be especially pertinent for PWV, as these entirely healthy resistance-training induced adaptations are likely to augment the propagation of soundwave transmission through the circulation and subsequently increase PWV. Of course, these healthy adaptations are completely different to the arteriosclerotic maladaptations that manifest during advanced ageing and disease, which also cause PWV to increase. Accordingly, elevated PWV in resistance-trained individuals is unlikely to reflect arterial disease, but rather, essential structural remodelling required to prevent wall rupture during acute bouts of intense resistance exercise. Therefore, making any inferences or associations between higher PWV and CVD risk in this population should be met with significant caution. In any event, PWV in the present study was similar between all three groups, displaying an average reading of 5.68 m.s^−1^, which represents the lowest 10th percentile for this age group and implies that the future risk of CVD in each group is comparably low (Mattace-Raso et al. 2010).

### The influence of training status on carotid artery 2D-strain parameters in response to isometric handgrip exercise

Both trained groups exhibited a dynamic reduction in PCS and S-SR and increase in β_2_ during the IHG protocol, which quickly returned to resting values during the recovery stage. In contrast, these 2D-Strain parameters were completely unresponsive during the entire IHG protocol in untrained men. These group differences in 2D-Strain responses were observed despite similar increases in CCA diameter and haemodynamics between the three groups. We have previously demonstrated that CCA diameter and haemodynamic variables are significant determinants of 2D-Strain parameters both at rest and in response to isometric leg press exercise in untrained men (Black et al. 2016a, b). However, these variables do not explain the training-induced differences in 2D-Strain responses to a lower-intensity IHG exercise challenge. Alternatively, the divergent responses in PCS and S-SR between both trained groups and the untrained group may reflect respective differences in the intrinsic properties of the arterial extracellular matrix.

Indeed, as systemic increases in pressure and loading occur during acute exercise, CCA diameter increases and extracellular control transfers to collagen fibres to maintain structural integrity (Wagenseil and Mecham 2009); consequently, reducing and decelerating arterial deformation. However, a persistent lack of exercise-induced extracellular fibre stimulation in untrained individuals may have desensitised collagen activation, possibly explaining the unchanged PCS and S-SR during the IHG protocol. Conversely, resistance-training is known to increase total collagen volume and sensitivity (Souza et al. 2017), and whilst endurance-training provokes greater elastin and reduced collagen volumes (Hanna et al. 2014), it can still elicit collagen fibre realignment and thereby improve cross-link efficiency, more effective recruitment, and greater vessel integrity (Tanaka et al. 2000). Therefore, trained groups may readily recruit collagen fibres during periods of increased pressure to ensure vessel stability and controlled distal perfusion, as reflected by an efficient transient reduction in PCS and S-SR during the IHG. Similarly, upon the cessation of exercise, CCA diameter and pressure return to resting values, which decreases the necessity for collagen recruitment, and elastin becomes primarily responsible for stress transfer across the CCA wall (Wagenseil and Mecham 2009); reflected by PCS and S-SR quickly returning to resting values in the trained groups. Despite the likely differences in the composition of the extracellular matrix, even between trained groups (Souza et al. 2017; Hanna et al. 2014), the volume of activated collagen fibres required for vessel stability during the IHG may be relatively low (Wagenseil and Mecham 2009). Therefore both endurance- and resistance-trained men likely have sufficient collagen volume to stabilise against low-intensity physiological stress and thereby express similar reductions in PCS and S-SR. Equally, the unresponsive untrained CCA may require higher loads and/or involvement of larger muscle groups to trigger similar 2D-Strain responses; a concept supported by our previous observation of acute reductions in PCS and S-SR in untrained young men during the more intensive isometric leg press (Black et al. 2016a, b).

Whilst the IHG exercise was a relatively low-intensity stimulus for the trained groups, it was still sufficient to evoke significant reductions in PCS and S-SR compared to the *unresponsive* untrained men, which may reflect a lower threshold at which the CCA responds to physiological stress. Importantly, these group differences in 2D-Strain responses were observed despite similar increases in *β*_1_ or *E*_p_ stiffness during IHG exercise between the three groups. Accordingly, 2D-Strain parameters provide novel and additional insight into global arterial wall stress mechanics during acute surges in pressure and CCA loading beyond that of conventional CCA stiffness indices. Although, to our knowledge, this is the first study to demonstrate that 2D-Strain imaging is more sensitive at detecting intricate training-specific differences in CCA responses during a physiological challenge; our findings build upon other previous reports that 2D-Strain is superior at detecting fitness- (Campbell et al. 2023; Pugh et al. 2018), age- (Bjallmark et al. 2010) and disease-related (Park et al. 2012; Kawasaki et al. 2009) changes in resting CCA stiffness than conventional methods.

Reductions in PCS and S-SR during the IHG in the trained groups may also represent a protective mechanism that facilitates more controlled and consistent blood flow to the cerebrovasculature, which might explain previous reports of improved cerebrovascular haemodynamics in both endurance (Tarumi et al. 2013) and resistance (Thomas et al. 2021) trained individuals. Unlike PCS and S-SR, D-SR reduced in all groups during IHG, however the magnitude of reduction was less in the endurance-trained group, most likely due to the greater reduction in PCS displayed by this group at the end of the protocol. Endurance-training is associated with greater regional cerebral blood flow at rest (Tarumi et al. 2013) and global cerebral blood flow during exercise (Ogoh and Ainslie 2009); therefore, it is plausible that under resting conditions and during lower exercise intensities, rapid diastolic recoil is unnecessary to maintain an already smooth blood flow to the cerebral circulation. Interestingly, a previous study reported no changes in PCS, S-SR or D-SR during an identical IHG protocol in either active or inactive middle-aged postmenopausal women, despite illustrating a positive correlation between 2D-Strain parameters and $\dot{V}{\text{o}}_{{{\text{2peak}}}}$ (Campbell et al. 2023). This indicates that any training-induced improvements in CCA sensitivity to a low-intensity loading stimulus in young individuals blunt with ageing, as extracellular matrix alterations that promote arterial stiffening manifest. In any event, the transient decrease in PCS and S-SR observed in both of our trained groups during IHG likely represent chronic training adaptations to help normalise wall stress and ensure consistent blood flow to the cerebrovasculature during acute exercise. Accordingly, both endurance- and resistance-trained men display comparable *dynamic* CCA responses to IHG that are superior to untrained counterparts, reinforcing our resting observations that resistance-trained men possess compliant and healthy CCAs.

### Limitations and future research

There are limitations to this study that should be acknowledged. First, we recognise that our findings are restricted to young male participants only. There are recognised sex differences in haemodynamic regulation and vascular function (Green et al. 2017), and therefore we acknowledge that resistance- and endurance-trained women may display different 2D-Strain values both at rest and in response to IHG exercise. Future studies that are adequately powered should explore the interaction between sex and training modality on 2D-Strain parameters. Second, our 2D-Strain findings are restricted to the CCA and therefore cannot be applied systemically. Peripheral muscular arteries might be more susceptible to endurance- (Dawson et al. 2008) and resistance-training (Kim et al. 2021) than elastic central arteries; therefore, 2D-Strain parameters should also be studied in peripheral vascular beds in the future. Third, we were unable to collect simultaneous measurements of CCA blood flow alongside 2D-Strain parameters. Although we have previously demonstrated that CCA blood flow has a negligible influence on 2D-Strain parameters at rest (Talbot et al. 2020), simultaneous blood flow and pulsatility measurements during the IHG protocol may have provided more mechanistic insight into the distinct 2D-Strain responses observed between the groups. We also recognise that attributing training-induced increases in PCS and S-SR to favourable extracellular matrix alterations is speculative, and acknowledge that other recognised training-induced adaptations including improvements in sympathetic adrenergic vasoconstrictor tone, endothelial function, and oxidative stress (Green et al. 2017) are also likely to influence CCA compliance. The interplay between these recognised exercise-induced vascular adaptions warrants further training-mode specific investigation. Finally, although training histories were acquired, we did not capture a detailed history of training intensity and therefore cannot discern the influence of overall training load. Dedicated longitudinal training interventions that adopt 2D-Strain imaging techniques are required to fully characterise the beneficial effects of resistance-training on central artery stiffness.

## Conclusion

To our knowledge, this is the first study to utilise 2D-Strain imaging to demonstrate that resistance- and endurance-trained men display comparable global CCA compliance that is superior to untrained men. In contrast, conventional PWV and *β*_1_ stiffness indices failed to differentiate between trained and untrained groups, reinforcing 2D-Strain as a more accurate technique to examine intricate training-specific alterations to the elastic properties of the arterial wall. Taken together, these findings indicate that both endurance- and resistance-training offer potent arterial benefit and contends previous reports that resistance-training increases central artery stiffness.

## Data Availability

Data supporting the study findings are available from the corresponding author upon reasonable request.

## Abbreviations

1RM: One repetition maximum
2D-Strain: Two-dimensional strain
ANCOVA: Analysis of covariance
ANOVA: Analysis of variance
CCA: Common carotid artery
CVD: Cardiovascular disease
D-SR: Diastolic strain rate
ECG: Electrocardiogram
*E*_p_: Petersons elastic modulus
IHG: Isometric handgrip
PWV: Pulse wave velocity
ROI: Region of interest
S-SR: Systolic strain rate
$\dot{V}{\text{o}}_{{{\text{2peak}}}}$: Peak oxygen consumption
*β*_1_: *β*_1_-Stiffness index
*β*_2_: *β*_2_-Stiffness index
